# Chemical Composition and Crystal Morphology of Epicuticular Wax in Mature Fruits of 35 Pear (*Pyrus* spp.) Cultivars

**DOI:** 10.3389/fpls.2018.00679

**Published:** 2018-05-23

**Authors:** Xiao Wu, Hao Yin, Zebin Shi, Yangyang Chen, Kaijie Qi, Xin Qiao, Guoming Wang, Peng Cao, Shaoling Zhang

**Affiliations:** ^1^Center of Pear Engineering Technology Research, State Key Laboratory of Crop Genetics and Germplasm Enhancement, Nanjing Agricultural University, Nanjing, China; ^2^Institute of Horticulture, Zhejiang Academy of Agricultural Sciences, Hangzhou, China

**Keywords:** wax, GC-MS, crystal morphology, PCA, pears

## Abstract

An evaluation of fruit wax components will provide us with valuable information for pear breeding and enhancing fruit quality. Here, we dissected the epicuticular wax concentration, composition and structure of mature fruits from 35 pear cultivars belonging to five different species and hybrid interspecies. A total of 146 epicuticular wax compounds were detected, and the wax composition and concentration varied dramatically among species, with the highest level of 1.53 mg/cm^2^ in *Pyrus communis* and the lowest level of 0.62 mg/cm^2^ in *Pyrus pyrifolia*. Field emission scanning electron microscopy (FESEM) analysis showed amorphous structures of the epicuticular wax crystals of different pear cultivars. Cluster analysis revealed that the *Pyrus bretschneideri* cultivars were grouped much closer to *Pyrus pyrifolia* and *Pyrus ussuriensis*, and the *Pyrus sinkiangensis* cultivars were clustered into a distant group. Based on the principal component analysis (PCA), the cultivars could be divided into three groups and five groups according to seven main classes of epicuticular wax compounds and 146 wax compounds, respectively.

## Introduction

Cuticular wax is the product of a complex mixture of very-long-chain (VLC) aliphatic compounds and their oxygenated derivatives, including fatty acids, alkanes, alcohols, esters, aldehydes, ketones, and triterpenes (Kolattukudy, 1996). It has been widely reported that wax plays important roles in moderating gas exchange, limiting non-stomatal water loss, protecting plants against ultraviolet (UV) radiation and extreme temperature damage, self-cleaning behavior and providing mechanical support to maintain the integrity of plant organs (Wang et al., 2016). More and more studies have suggested that the fruit cuticular wax layer acts as the first protective barrier against fruit splitting and plays pivotal roles in the reduction of pathogenic and insect attacks, protection against mechanical damage (Chu et al., 2017). Studies also showed that cuticular wax play important roles in maintaining the postharvest quality and delaying fruit senescence. For example, the removal of the natural wax of blueberry fruits can accelerate the postharvest water loss and decay, reduce the sensory and nutritional qualities, and then shorten the fruit shelf-life (Chu et al., 2018). In addition, as one of the most important quality factors determining consumer demand, the apple’s appearance during the postharvest storage were also determined by the physical and chemical properties of wax composition (Glenn et al., 1990).

The chemical composition and morphology of the cuticular wax layer of several fruit species have been detected. For example, triterpenoids and β-diketones were the most prominent compounds in blueberry fruits, and a large amount of tubular wax was deposited on their surfaces (Chu et al., 2017). In the mature fruits of olives, triterpenes were the main compounds, and only epicuticular waxes were observed, arranged in various crystalloid structures, including granules, platelets, plates and rodlets (Lanza and Di Serio, 2015). Aldehydes, triterpenes and secondary alcohols were the most predominant components of cuticular wax from citrus and plum fruits (Ismail et al., 1977; Wang et al., 2014). The wax concentration, chemical composition and morphology were also reported to be positively correlated with disease resistance capabilities (Wu et al., 2017). Therefore, a clear understanding of the components and amounts of fruit wax is important for obtaining better fruit quality, improving disease resistance and developing postharvest treatment strategies. More importantly, the structure, composition and concentration of fruit wax vary among cultivars of the same species; thus, knowledge of the cuticular wax traits from different germplasms will also be helpful for the selection of breeding parents. To the best of our knowledge, the cuticular wax profiles of different cultivars from several fruit and vegetable species have been detected, such as various cultivars of apple (Belding et al., 1998), grape (Pensec et al., 2014), blueberry (Chu et al., 2017), persimmon (Tsubaki et al., 2012), tomato (Bauer et al., 2004), and pepper (Parsons et al., 2012).

As the third most important temperate fruit species, the pear belongs to the Rosaceae family. The *Pyrus* genus is genetically diverse with 1000s of cultivars, which can be divided into two major groups, Occidental (European) and Oriental (Asiatic) pears. The Oriental pears comprise *Pyrus bretschneideri*, *Pyrus ussuriensis*, *Pyrus pyrifolia*, and *Pyrus sinkiangensis* and are mainly grown in China, Korea, Japan, and other Asian countries, whereas the Occidental pear (*Pyrus communis* Linn.) is mainly produced in America, Italy, Spain, and Germany. Previous studies have reported that the amount of wax obtained from four pear varieties (‘Pingguoli’ and ‘Xuehua’, *P. bretschneideri*; ‘Kuerle’, *P. sinkiangensis*; ‘Yuluxiang’, hybrid cultivars) varied dramatically, with wax concentrations ranging from 0.32 mg/cm^2^ (‘Pingguoli’) to 1.43 mg/cm^2^ (‘Kuerle’) (Chen et al., 2014; Wu et al., 2017); however, in the mature fruit of pears belonging to the other three species, information about the epicuticular wax profile is very limited. More generally, the genetic relationships of several important fruit-related traits of pear have been reported, such as sugar, acid, and flesh color (Liu et al., 2016; Wu et al., 2014), but genetic relationship of pear cuticular wax was less reported. Thus, the investigation of the pear cuticular wax at the germplasm level is of significant value for both the genetic study and the higher wax pear breeding program.

In this study, 35 pear cultivars belonging to five different species and hybrid interspecies were selected for determination of the concentration, composition, structure and genetic relationship of epicuticular wax through GC–MS, FESEM and cluster analysis. The results in the present study will not only help further studies of the potential role of wax components on postharvest quality but also provide a foundation for plant breeding aimed at improving fruit epicuticular wax.

## Materials and Methods

### Plant Material

The composition, concentration and structure of epicuticular wax compounds of the fruits of 32 pear cultivars (**Table 1**) were evaluated in 2016, and the data of other three pear cultivars (‘Kuerle’, ‘Xuehua’ and ‘Yuluxiang’) was originated from our previous research (Wu et al., 2017). The fruits at commercial maturity, without disease, infection or physical injuries, were selected for the following experiments. Thirty fruits of each cultivar were packed individually in a plastic net bag and delivered immediately to the laboratory at Nanjing Agricultural University. All the samples were examined immediately.

**Table 1:** Cultivars of pears used in the study.

Species (parentage)		Code	Cultivars	Origin place	Sample place	Sampling time	Tree-ages and management style	Provide unit
*Pyrus communis* Linn.		1	Docteur Jules Guyot	France	Shandong	2016.8.3	8–10-year-old tree; Routine management	Yantai Academy of Agricultural Sciences
		2	Clapp Favorite	America	Shandong	2016.8.10		Yantai Academy of Agricultural Sciences
		3	Abbe Fetel	France	Shandong	2016.9.11		Yantai Academy of Agricultural Sciences
		4	Red Clapp Favorite	America	Shandong	2016.8.7		Yantai Academy of Agricultural Sciences
		5	Bartlett Max Red	America	Shandong	2016.9.11		Yantai Academy of Agricultural Sciences
*Pyrus ussuriensis* Maxim.		6	Tianjianba	Liaoning	Liaoning	2016.9.4		Liaoning Institute of Fruit Sciences
		7	Hongnanguo	Liaoning	Liaoning	2016.9.6		Liaoning Institute of Fruit Sciences
		8	Balixiang	Liaoning	Liaoning	2016.9.15		Chinese Academy of Agricultural Sciences
		9	Nanguoli	Anshan, Liaoning	Liaoning	2016.9.6		Liaoning Institute of Fruit Sciences
		10	Huagai	Liaoning	Liaoning	2016.9.4		Liaoning Institute of Fruit Sciences
*Pyrus sinkiangensis* Yü.		11	Kuerle	Korla, Xinjiang	Xinjiang	2015.9.9		Research Center of Korla Fragrant Pear
		12	Kalaamute	Xinjiang	Xinjiang	2016.9.12		Research Center of Korla Fragrant Pear
		13	Kuikeamute	Xinjiang	Xinjiang	2016.9.10		Research Center of Korla Fragrant Pear
		14	Lüamute	Xinjiang	Xinjiang	2016.9.13		Research Center of Korla Fragrant Pear
		15	Kucheamute	Xinjiang	Xinjiang	2016.9.10		Research Center of Korla Fragrant Pear
*Pyrus bretschneideri* Rehd.		16	Qiubai	Hebei	Hebei	2016.9.5		Hebei Academy of Agriculture and Forestry Sciences
		17	Pingguoli	Yanbian, Jilin	Liaoning	2016.9.18		Liaoning Institute of Fruit Sciences
		18	Eli	Zhejiang	Nanjing	2016.7.15		Nanjing Agricultural University
		19	Xuehuali	Dingxian, Hebei	Hebei	2015.9.4		Hebei Academy of Agriculture and Forestry Sciences
		20	Chili	Chiping, Shandong	Shandong	2016.9.22		Yantai Academy of Agricultural Sciences
*Pyrus pyrifolia* BurmNakai.		21	Shinseiki	Japan	Nanjing	2016.7.15		Nanjing Agricultural University
		22	Kousui	Japan	Nanjing	2016.7.18		Nanjing Agricultural University
Hybrid cultivars	Pingguoli × Chili	23	Jinfeng	Xingcheng, Liaoning	Liaoning	2016.9.4		Liaoning Institute of Fruit Sciences
	Xizilü × Cuiguan	24	Cuiyu	Zhejiang	Nanjing	2016.7.15		Nanjing Agricultural University
	Kuerle × Eli	25	Hongxiangsu	Zhengzhou, He’nan	Nanjing	2016.9.3		Nanjing Agricultural University
	Kuerle × Xuehua	26	Yuluxiang	Taigu, Shanxi	Nanjing	2015.9.7		Shanxi Academy of Agricultural Sciences
	Pingguoli × Mishirazu	27	Zaosu	Xingcheng, Liaoning	Nanjing	2016.7.24		Nanjing Agricultural University
	Shinseiki × Xuehua	28	Zhongli No. 2	Zhengzhou	Zhengzhou	2016.8.16		Zhengzhou Fruit Research Institute
	Xizilü × Cuiguan	29	Chuxialü	Zhejiang	Zhejiang	2016.7.15		Zhejiang Academy of Agricultural Sciences
	Kuerle × Zaosu	30	Xinli No. 7	Xinjiang	Nanjing	2016.7.15		Nanjing Agricultural University
	Shinseiki × (Bayun × Hangqing)	31	Xizilü	Zhejiang	Zhejiang	2016.8.5		Zhejiang Academy of Agricultural Sciences
	Kousui × (Hangqing × Shinseiki)	32	Cuiguan	Zhejiang	Nanjing	2016.7.24		Nanjing Agricultural University
	Xuehua × Shinseiki	33	Xueqing	Zhejiang	Zhejiang	2016.8.14		Zhejiang Academy of Agricultural Sciences
	? × Chili	34	Hangqing	Zhejiang	Zhejiang	2016.8.5		Zhejiang Academy of Agricultural Sciences
	Xuehua × Shinseiki	35	Huangguan	Hebei	Nanjing	2016.7.25		Nanjing Agricultural University

*The data of ‘Kuerle’, ‘Xuehua’ and ‘Yuluxiang’ was originated from our previous research (Wu et al., 2017).*

### Extraction of the Epicuticular Wax

The epicuticular wax was extracted according to the method of Li et al. (2014). After being washed with tap water and air dried, three groups of five pear fruits were fully immersed and agitated twice for 1 min in 600 mL chloroform under a fume hood. The solvent containing the waxes was transferred into pre-weighed vials and evaporated by a nitrogen-blowing instrument (JHD-001S, Shanghai Jiheng Industries Company, Ltd.) at 40°C, and the wax weights were recorded.

### Determination of the Epicuticular Wax Concentration

The surface area of the pear fruits was calculated according to the method of Yin et al. (2011). The wax concentration (μg/cm^2^) was calculated with the following formula:

$$\text{Wax concentration}=({\text{W}}_{\text{1}}-{\text{W}}_{0})/\text{Sa}$$

where W_1_ is the final weight of the vials (μg); W_0_ is the initial weight of the vials (μg); and Sa is the total surface area of 5 pear fruits (cm^-2^).

### Chemical Analysis by GC-MS

The components present within each wax extraction prepared were analyzed using the method of Li et al. (2014). The wax extraction (1 mg) dissolved with 1.2 mL chloroform was carried out on a Bruker 450-GC, coupled with a Bruker 320-MS and a BR-5MS capillary column (30 m FS, 0.25 μm ID, 0.25 μm df). Helium was used as a carrier gas at a flow rate of 1.2 mL min^-1^. The following parameters were employed: inlet temperature, 280°C; MS transfer line temperature, 280°C; ion source temperature, 250°C; quadrupole temperature, 150°C; electron impact (EI), 70 eV; and m z^-1^ range, 50–650.

GC was carried out at the following temperature settings. First, the temperature was set to 50°C for 2 min. Next, it was increased to 200°C at a rate of 40°C min^-1^ and held at this temperature for 2 min. Finally, it was increased to 320°C at a rate of 3°C min^-1^ and held at this temperature for 30 min.

### Electron Microscope Observations

The epicuticular wax was observed by FESEM according to the method of Wu et al. (2017). Pericarp pieces (3 × 3 × 1 mm) from the equatorial sections of three fruits for each cultivar were excised using a blade, fixed in 2.5% glutaraldehyde. The dehydrated samples were attached to a sample stage with conductive tape and coated with gold particles using a Hitachi E-1045. The coated samples were examined using a Hitachi 4800 field emission scanning electron microscope.

### Statistical Analysis

The chemical composition of epicuticular wax was analyzed using the NIST 2013 Library. Box-plot, principal component analysis (PCA) and heatmap analysis of the dataset for wax concentration and composition were used to detect clustering and to establish relationships; these analyses were carried out using the R programming language. For the heatmap and clustering analysis, the scaled data file with Log transformed was calculated, and analyzed by the ‘complete’ algorithm in the heatmap.2 package (Murtagh and Legendre, 2014). We performed the principal component analysis (PCA) by the algorithm of ‘Multivariate Analysis’ in the ‘prcomp’ module of the R package, and 3D plot of PCA was drawn by the scatterplot3d packages (Mardia et al., 1979). The SPSS20 statistical software package (IBM Software Group) was used for all statistical analysis. Data were compared by Student’s *t*-test, and significant differences are marked with different letters (*P* < 0.05). Data are shown as the mean ± SD (*n* = 3).

## Results and Discussion

### Wax Amounts and VLC Aliphatic Compounds

**FIGURE 1:**
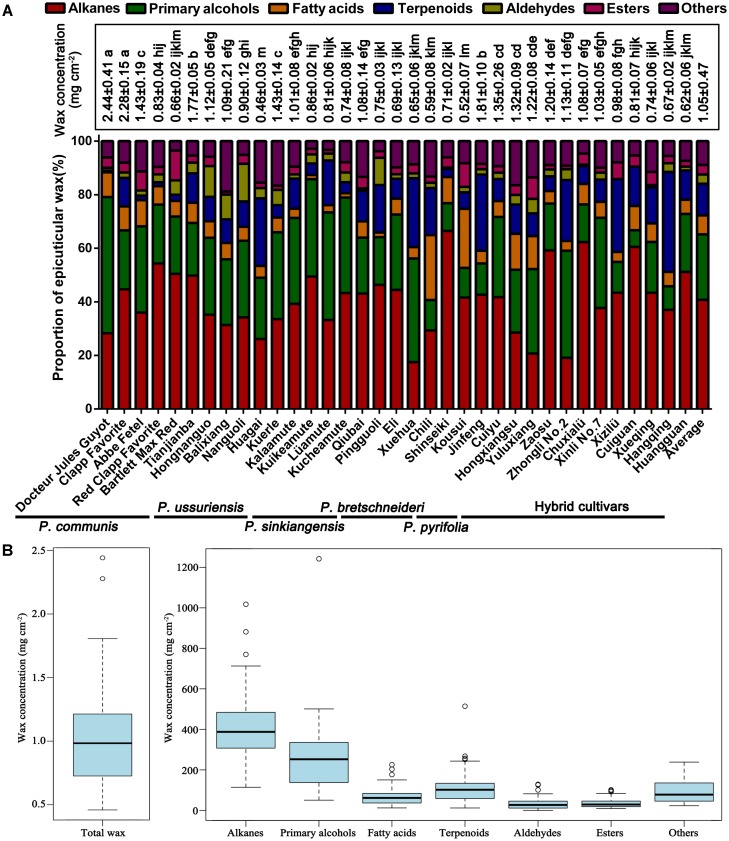
Amounts and compositions of epicuticular wax in the mature fruits of 35 pear cultivars. **(A)** Total epicuticular wax concentration and relative wax compositions of the mature fruits of 35 pear cultivars. Statistical significance was determined using Student’s *t*-test. Each value is the mean ± SD of three biological repeats. Different letters above each bar indicate significant differences at the 5% level. **(B)** Range and distribution of total wax and seven wax compounds in 35 pear cultivars. The horizontal lines in the interior of the box are the median values. The height of the box is equal to the interquartile distance, indicating the distribution for 50% of the data. Approximately 99% of cultivars fall inside the whiskers, and the cultivars outside these whiskers are considered outliers or extreme values, indicated by horizontal lines and circles.

The total wax of mature fruits from 35 pear cultivars was collected via chloroform extraction (**Table 1**). The amount of wax obtained from the 35 pear varieties varied dramatically (**Figure 1** and Supplementary Tables S1, S2), and the wax concentrations ranged from 0.46 ± 0.03 mg/cm^2^ (‘Huagai’, *P. ussuriensis*) to 2.44 ± 0.41 mg/cm^2^ (‘Docteur Jules Guyot’, *P. communis*) (**Figure 1A**). On the species level, the epicuticular wax concentration was the highest in *P. communis* (1.53 ± 0.77 mg/cm^2^) and the lowest in *P. pyrifolia* (0.62 ± 0.12 mg/cm^2^) (Supplementary Table S2). Similar results have been identified among other species, such as pepper (2.15–7.52 μg/cm^2^) (Parsons et al., 2012), persimmon (337–770 μg/cm^2^) (Tsubaki et al., 2012), tomato (27–79 μg/cm^2^) (Bauer et al., 2004), blueberry (48–172 μg/cm^2^) (Chu et al., 2017), grape (3.5–5.5 mg/g) (Pensec et al., 2014) and apple (366–1038 μg/cm^2^) cultivars (Belding et al., 1998). Compared with these species, the average wax concentration of pears (1.05 mg/cm^2^) was the second highest, lower than only that of grapes. Various methods of wax extraction and surface area calculation were employed in these species; thus, the considerable variation of epicuticular wax obtained from different species was reasonable. In total, 146 epicuticular wax compounds were detected in the fruits of 35 cultivated pear species (Supplementary Table S1 and Supplementary Figure S1), mainly primary alcohols (26 compounds), alkanes (25 compounds), fatty acids (25 compounds), terpenoids (23 compounds), esters (16 compounds) and aldehydes (8 compounds), and some other small amounts of compounds (23 compounds, Supplementary Table S1); the content of these wax compounds accounted for 24.47, 40.72, 7.09, 11.80, 3.59, 3.45, and 8.88% of the total content, respectively (**Figure 1A**). Although the primary alcohols comprised the highest number of wax compounds, the alkanes were the dominant compounds in the epicuticular wax of pear fruits in terms of amounts. It has also been reported that alkanes are the major wax component in many fruits, including tomato (Leide et al., 2007), mandarin (Sala, 2000) and grapefruit (McDonald et al., 1993). However, limited studies reported that primary alcohols are essential components in fruits. Similar to the total wax content, the amount of each component also varied among the five different species and hybrid interspecies (Supplementary Figure S2). For example, the concentrations of alkanes, primary alcohols, fatty acids, esters and other epicuticular wax compounds were much higher in *P. communis* than in the other four species and hybrid interspecies (Supplementary Figures S2B,D,F,L,N), whereas the hybrid cultivars and *P. ussuriensis* had the highest concentrations of terpenoids and aldehydes, respectively (Supplementary Figures S2H,J). Similar results were also reported in blueberry fruits from nine blueberry cultivars belonging to three different species (Chu et al., 2017).

**FIGURE 2:**
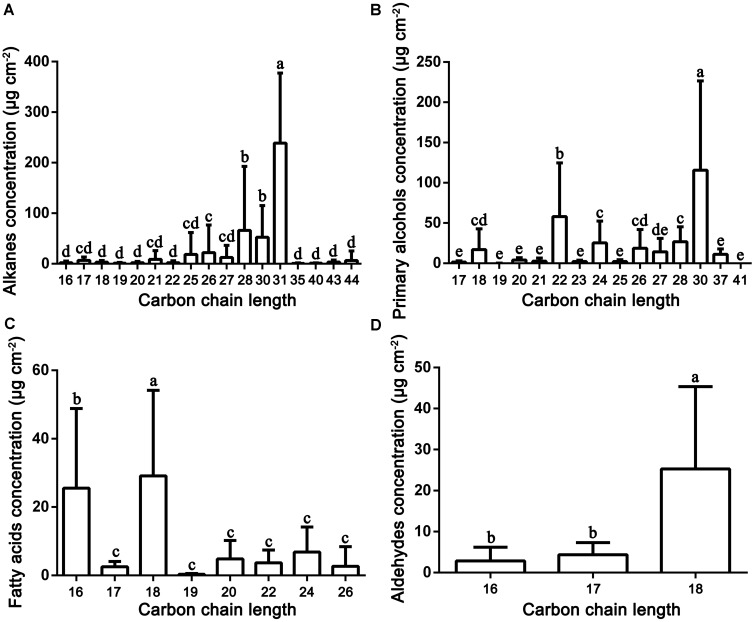
Distribution and concentration of the main VLC aliphatic compounds detected in the epicuticular wax of 35 pear cultivars. **(A)** Alkanes; **(B)** primary alcohols, **(C)** fatty acids; and **(D)** aldehydes. Statistical significance was determined using Student’s *t*-test. Each value is the mean ± SD of three biological repeats. Different letters above each bar indicate significant differences at the 5% level.

Very-long-chain aliphatic compounds were the major epicuticular wax components found in the fruit surfaces of mature pears. Abundant VLC aliphatic compounds were detected in the 35 pear cultivars, and their concentrations varied greatly, ranging from 336.85 μg/cm^2^ (‘Huagai’, *P. ussuriensis*) to 769.87 μg/cm^2^ (‘Kuikeamute’, *P. sinkiangensis*) (Supplementary Table S2). Our results also showed that the VLC aliphatic compounds of pear fruits are mainly composed of alkanes (C_16_–C_44_), primary alcohols (C_17_–C_41_), fatty acids (C_16_–C_26_), and aldehydes (C_16_–C_18_) (**Figure 2**).

Of all the detected 35 pear cultivars, 20 (10 hybrid combinations) were genetically related (Supplementary Figure S3). This was designed to further understand the regular relationship of the epicuticular wax between the hybrids and their parents. Our results showed that five hybrid cultivars (‘Hongxiangsu’, ‘Yuluxiang’, ‘Xinli No. 7’, ‘Chuxialü’ and ‘Cuiyu’) with higher concentration were consistent with that of their parents (Supplementary Figures S3A–D), other three hybrid cultivars (‘Huangguan’, ‘Xueqing’ and ‘Cuiguan’) were also similar with their parents obtained lower concentration of epicuticular wax (Supplementary Figures S3G,H), and only two hybrid cultivars (‘Jinfeng’ and ‘Zhongli No. 2’) with higher concentration were contrary with that of their parents (Supplementary Figures S3E,F). These indicate that the epicuticular wax concentration of hybrid pear cultivars was positively correlated with that of the parents. Take an example, ‘Kuerle’ (*Pyrus sinkiangensis* Yü.) with relatively higher wax concentration is the most famous pear cultivar in China, not only for its pleasant appearance, aroma and taste, but also the longer postharvest storage period. Here, three hybrid cultivars (‘Hongxiangsu’, ‘Yuluxinag’ and ‘Xinli No. 7’) have all inherited the higher wax concentration from ‘Kuerle’, making it an excellent parent for pear breeding. In addition, ‘Docteur Jules Guyot’ (*P. communis*), ‘Tianjianba’ (*P. ussuriensis*), ‘Qiubai’ (*P. bretschneideri*) and ‘Shinseiki’ (*P. pyrifolia*) should be the corresponding optimal parent cultivars in each species for the pear breeding with higher wax concentrations.

#### Alkanes

Alkanes are important VLC aliphatic compounds in the epicuticular wax of pear fruits, with concentrations ranging from 114.19 μg/cm^2^ (‘Xuehua’, *P. bretschneideri*) to 1017.49 μg/cm^2^ (‘Clapp Favorite’, *P. communis*), respectively (**Figure 1A** and Supplementary Figure S2A). On the species level, the alkane concentrations were the highest in *P. communis* (601.14 μg/cm^2^), followed by hybrid cultivars (442.75 μg/cm^2^), *P. ussuriensis* (408.77 μg/cm^2^), *P. sinkiangensis* (578.50 μg/cm^2^) and *P. pyrifolia* (408.77 μg/cm^2^), but they were the lowest in *P. bretschneideri* (281.72 μg/cm^2^) (Supplementary Figure S2B). Many studies have shown that alkanes are predominant in epicuticular wax on many fruits, such as mandarin (148–234 mg/g) (Sala, 2000), pepper (1.4–47.4 μg/dm^2^) (Parsons et al., 2012) and tomato (55%, 13.75 μg/cm^2^) (Bauer et al., 2004); leaves, including those of *Schefflera elegantissima* (∼80%, 400 μg/cm^2^), *Garcinia spicata* (∼72%, 864 μg/cm^2^) (Jetter and Riederer, 2016), castor bean (64.1%–70.7%, 88.1–139.4 μg/cm^2^) (de Araújo Silva et al., 2017) and aloe (34.4%, 8.8 μg/cm^2^) (Racovita et al., 2015); and both fruits and leaves of cucumber (46.1%, 59.6 μg/cm^2^; 61.9%, 110.3 μg/cm^2^) (Wang W. et al., 2015). Compared with these species, the average wax concentration of pears (422.73 μg/cm^2^) is the second highest, only lower than that of *Garcinia spicata*. VLC alkanes ranging from hexadecane (C_16_) to tetratetracontane (C_44_) were detected in the fruit wax of the 35 pear species, and C_28_ to C_31_ were the most abundant. For example, the average amount of hentriacontane (C_31,_ 238.94 μg/cm^2^) was 3.70 times higher than the amount of the least abundant alkane (**Figure 2A**). Similarly, hentriacontane (C_31_) was also detected as the predominant alkane in cuticle wax of ‘Newhall’ navel orange (Wang et al., 2014), tomato (Bauer et al., 2004), pepper, eggplant (Bauer et al., 2005) and aloe (Racovita et al., 2015). It has also been reported that alkanes are associated with limiting water loss (Zhang et al., 2007). Furthermore, low temperature induced the alkane-forming pathway resulting in the accumulation of very long chain alkanes (including C22, C27, C29, C31 alkane) and their derivatives on the surface of apple fruit peel, which may be positive response to the environment signals of temperature and extension of the storage period (Hao et al., 2017). Thus, the higher proportion of alkanes in epicuticular wax of pear fruits may play a significant role in limiting water loss and prolonging the shelf-life to keep the fruits juicy.

#### Primary Alcohols

Twenty-six primary alcohols were detected as the second dominant component in the epicuticular wax of pear fruits, accounting for 24.47% of the total wax (**Figure 1A** and Supplementary Table S1). The concentrations of primary alcohols in the epicuticular wax of pear fruits ranged from 50.54 μg/cm^2^ (‘Cuiguan’, hybrid cultivars) to 1242.11 μg/cm^2^ (‘Docteur Jules Guyot’, *P. communis*), and the average concentration was 271.04 μg/cm^2^ (**Figure 2C**). On the species level, the primary alcohols were the highest in *P. communis* (505.53 μg/cm^2^), followed by *P. sinkiangensis* (337.77 μg/cm^2^), *P. ussuriensis* (259.60 μg/cm^2^), hybrid cultivars (228.15 μg/cm^2^) and *P. bretschneideri* (174.87 μg/cm^2^), but the primary alcohol levels were the lowest in *P. pyrifolia* (65.75 μg/cm^2^) (Supplementary Figure S2D). Higher concentrations of primary alcohols have been detected only in the epicuticular wax of leaves, such as wheat (42.5–72.9%) (Wang Y. et al., 2015), salix (32–51%) (Teece et al., 2008) and citrus plants (23.0–38.4%) (Baker et al., 1975). By contrast, the epicuticular wax of fruits with lower concentrations of primary alcohols have been reported in several species, such as 3.2% in blueberries (Chu et al., 2017), 0–9.1% in apples (Belding et al., 1998) and 6.0–15.0% in citrus fruits (Baker et al., 1975), respectively. A wide distribution of VLC primary alcohol homologues ranging from C_17_ to C_41_ was detected in the wax of pear fruits. As shown in **Figure 2B** and Supplementary Table S1, the C_30_ alcohols, including triacontanol and triacontane-1,30-diol (average 115.66 μg/cm^2^), were the most abundant primary alcohols; a similar result has been reported in the epicuticular wax of blueberry fruits (Chu et al., 2017). In addition, tetracosanol (C_24_), dotriacontanol (C_32_), and octacosanol (C_28_) were the dominant primary alcohols in the fruit wax of lemons (Baker et al., 1975) and tomatoes (Bauer et al., 2004) and the leaf wax of wheat (Wang Y. et al., 2015), respectively, whereas hexacosanol (C_26_) has been detected as the main primary alcohol in the leaf wax of both orange (Baker et al., 1975) and salix plants (Teece et al., 2008).

#### Fatty Acids

Sixteen VLC fatty acids ranging from C_16_ to C_26_ were detected in the fruit wax of 35 pear species. The highest concentration of fatty acids was observed in ‘Docteur Jules Guyot’ (*P. communis*, 225.27 μg/cm^2^), whereas the lowest was detected in ‘Kucheamute’ (*P. sinkiangensis*, 12.28 μg/cm^2^), and the average concentration was 74.44 μg/cm^2^ (Supplementary Figure S2E). On the species level, the fatty acids were the highest in *P. communis* (132.95 μg/cm^2^), followed by *P. pyrifolia* (92.00 μg/cm^2^), hybrid cultivars (74.31 μg/cm^2^), *P. ussuriensis* (67.48 μg/cm^2^), and *P. bretschneideri* (57.99 μg/cm^2^), but they were the lowest in *P. sinkiangensis* (32.66 μg/cm^2^) (Supplementary Figure S2F). The fatty acid profile was predominantly composed of C_16_ and C_18_ carbons, and a similar result has been reported in the epicuticular wax of peach fruits (**Figure 2C**) (Belge et al., 2014). Fatty acids with moderate concentrations, being a common component, were widespread in the epicuticular wax. Compared with pears (C_16_–C_26_), the carbon chain length of fatty acids in other fruit species (C_16_–C_34_) is longer, and the prominent components of fatty acids are more than 20 carbon atoms. For example, the fatty acid profiles in fruits of blueberry (C_16_–C_30_) (Chu et al., 2017) and lemon (C_16_–C_34_) were predominantly composed of C_30_ chains, and the dominant fatty acids in fruits of orange (C_16_–C_34_), clementine (C_16_–C_32_) and mandarin (C_16_–C_28_) consisted of 28 carbon atoms, while in the leaves of these three citrus species (all C_16_–C_34_), chains with 32 carbon atoms were the major fatty acid component (Baker et al., 1975).

#### Aldehydes

Aldehydes were found in all cultivars but at low concentrations in the epicuticular wax of pear fruits, accounting for 3.45% of the total wax (**Figure 1A**). The highest concentration of aldehydes was observed in ‘Hongnanguo’ (*P. ussuriensis*, 128.69 μg/cm^2^), whereas it was not detected in ‘Cuiguan’ (hybrid cultivars), and the average concentration was 35.32 μg/cm^2^ (Supplementary Figure S2I). On the species level, the aldehydes were the highest in *P. ussuriensis* (87.17 μg/cm^2^), followed by *P. sinkiangensis* (35.04 μg/cm^2^), *P. communis* (32.00 μg/cm^2^), hybrid cultivars (25.35 μg/cm^2^) and *P. bretschneideri* (24.40 μg/cm^2^), and they were the lowest in *P. pyrifolia* (6.84 μg/cm^2^) (Supplementary Figure S2J). A series of aldehydes composed of 16, 17, and 18 carbon atoms were detected in pear wax, and octadecanal, 9-octadecenal,(Z)- and 13-octadecenal,(Z)- (C_18_, average 25.87 μg/cm^2^) was the most abundant aldehyde (**Figure 2D** and Supplementary Table S1). However, similar to the fatty acids, the carbon chain length of aldehydes was longer in other species than in pears, and the concentration and main type of aldehydes in different species showed considerable variation. The aldehydes were the main components in the total wax of citrus fruits including ‘Satsuma’ (C_16_–C_32_, 45% of the total wax) and ‘Newhall’ navel orange (C_16_–C_33_, 46% of the total wax) fruits (Wang et al., 2014). Aldehydes comprising 24 and 32 carbon atoms were found in olive wax and accounted for the smallest proportion (3–5%) of total wax (Bianchi et al., 1992). As direct precursors of alkanes, aldehydes determine the alkane formation. Similar to alkanes, aldehydes have been identified as playing an important role in avoiding water loss (Wang et al., 2014).

#### Esters

Similar to aldehydes, the proportion of esters accounted for 3.59% of the total wax. The highest concentration of esters was observed in ‘Abbe Fetel’ (*P. communis*, 101.63 μg/cm^2^), whereas the lowest was detected in ‘Huagai’ (*P. ussuriensis*, 9.34 μg/cm^2^), and the average concentration was 36.77 μg/cm^2^ (Supplementary Figure S2K). On the species level, the ester concentrations were the highest in *P. communis* (75.01 μg/cm^2^) and the lowest in *P. sinkiangensis* (21.78 μg/cm^2^) (Supplementary Figure S2L). It is noteworthy that 1-monopalmitin and 1-monostearin were the only two glycerides compounds simultaneously detected in all 35 pear cultivars in this study (Supplementary Table S1). Glycerides were interesting ester compounds which have been rarely detected in waxes, till now, they have only been detected in the cuticular waxes of potato tuber and olives fruits (Vichi et al., 2016; Guo and Jetter, 2017). In addition, the epicuticular wax of fruits, esters were also detected as being the smallest proportion of the total wax in many species, including plums (8.2%) (Ismail et al., 1977), grapes (<5.1%) (Casado and Heredia, 1999) and apple fruits (1.39–4.85%) (Veraverbeke et al., 2001), while esters were not detected in blueberry (Chu et al., 2017) or peach fruits (Belge et al., 2014).

### Terpenoids

In addition to aliphatic compounds, a total of 23 terpenoid compounds, accounting for 11.80% of the total wax, were detected in the epicuticular waxes of the 35 pear cultivars. The highest concentration of terpenoids was observed in ‘Jinfeng’ (hybrid cultivars, 514.02 μg/cm^2^), whereas the lowest was detected in ‘Red Clapp Favorite’ (*P. communis*, 14.96 μg/cm^2^), and the average concentration was 36.77 μg/cm^2^ (Supplementary Figure S2G). On the species level, the terpenoids were highest in hybrid cultivars (165.91 μg/cm^2^), followed by *P. ussuriensis* (118.62 μg/cm^2^), *P. bretschneideri* (115.77 μg/cm^2^), *P. sinkiangensis* (76.25 μg/cm^2^) and *P. communis* (62.58 μg/cm^2^) and the lowest in *P. pyrifolia* (26.89 μg/cm^2^) (Supplementary Figure S2H).

Terpenoids was the predominant component of epicuticular wax in many species, such as the blueberry (64.2%) (Chu et al., 2017), grape (34–49%) (Pensec et al., 2014), peach (44.05–51.92%) (Belge et al., 2014) and sweet cherry (75.6%) (Peschel et al., 2007). Similar to the fruit wax of the tomato (Bauer et al., 2004), grapefruit (Nordby and McDonald, 1994) and citrus (Wang et al., 2014) and the leaf wax of eucalyptus (Viana et al., 2010), we detected lanosterol, farnesene, amyrin, lupeol, squalene, sitosterol and urs-12-en-28-al in the epicuticular wax of pear fruits (Supplementary Table S1). It has been reported that the accumulation of farnesene in the cuticular waxes of apple was the significant cause of greasiness (Christeller and Roughan, 2016). Several certain pear cultivars, such as ‘Yuluxiang’, ‘Kuerle’ and ‘Hongxiangsu’, developing a greasy surface, have accumulated more fluid wax constituents, which were mainly of farnesene. Although ursolic acids and oleanolic acids are the prominent terpenoid components in the cuticular waxes of apple (32.03–69.8%) (Belding et al., 1998), blueberry (16.4–26.0%) (Chu et al., 2017), peach (17.21–29.19%) (Belge et al., 2014), plum (1.0–5.4%) (Ismail et al., 1977), and sweet cherry (7.5–60.0%) (Peschel et al., 2007), they were not detected in epicuticular wax of pear fruits. In addition to the common terpenoid compounds, the pear fruits also contained specific compounds, such as 20α-dihydro pregnenolone (0–7.77 μg/cm^2^) (Supplementary Table S1).

Terpenoids are of significant importance for maintaining the mechanical strength of the cuticular membrane in persimmon fruit, as well as for defending against biotic and abiotic stress, delaying fruit senescence and being involved in important biological activities. Here, we detected four types of tocopherols in 30 cultivated pear species (except ‘Hongnanguo’, ‘Huangguan’, ‘Nanguo’, ‘Docteur Jules Guyot’ and ‘Kousui’), including delta-tocopherol, beta-tocopherol, gamma-tocopherol and alpha-tocopherol (Supplementary Table S1). As promising sources of biological activities, all four types of tocopherols were simultaneously detected in the mature fruits of ‘Xizilü.’ Tocopherol plays important roles in inhibiting the growth of pathogens and protecting fruits against biotic stresses, and squalene may extend the storage time by removing free radicals and enhancing oxygen loading in citrus fruits (Wang et al., 2014; Wu et al., 2017). Tocopherol and squalene concentrations were 0–81.07 μg/cm^2^ and 0–7.34 μg/cm^2^ in the epicuticular waxes of the 35 pear cultivars (Supplementary Table S1). Prior studies have noted that tocopherol and squalene can lead to enhanced resistance to *Alternaria* rot in pear fruits (Wu et al., 2017).

### Crystal Morphology

**FIGURE 3:**
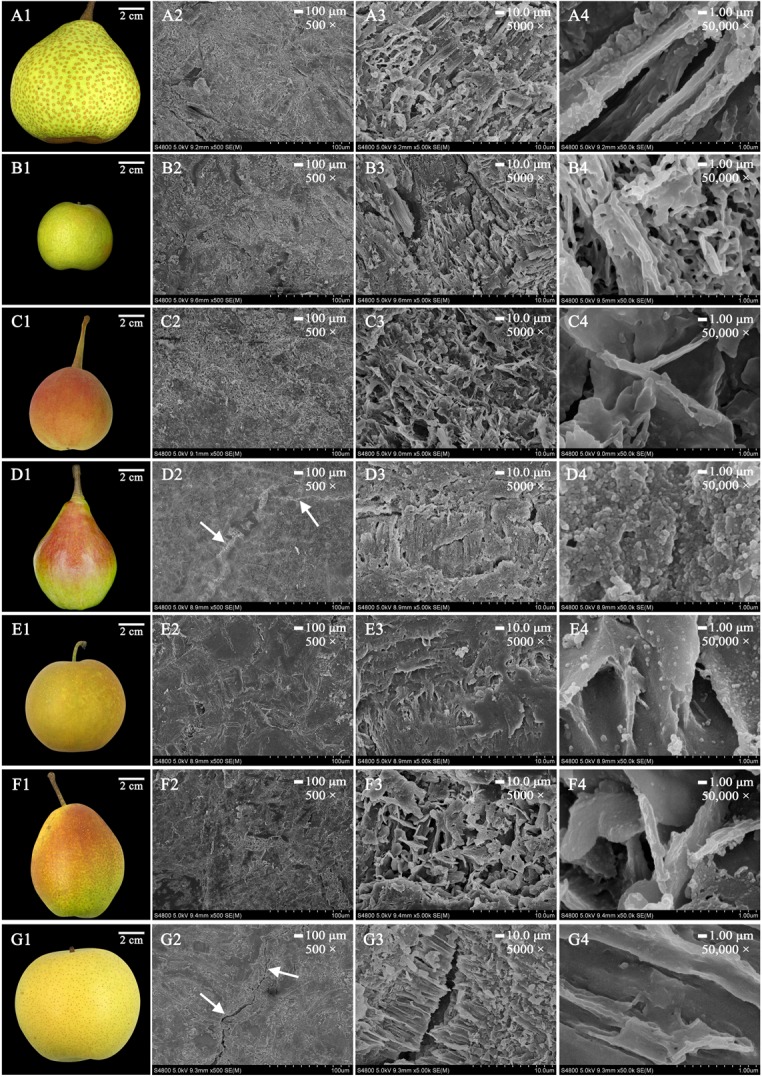
The morphology of mature fruits and the magnification series of the FESEM images of epicuticular wax in seven pear cultivars. **(A1–G1)** Are mature fruits of ‘Chili’, ‘Nanguoli’, ‘Kucheamute’, ‘Clapp Favorite’, ‘Shinseiki’, ‘Hongxiangsu’ and ‘Zhongli No. 2’, respectively. Scale bars represent 100 μm (magnification 500×) in **(A2–G2)**, 10.0 μm (magnification 5000×) in **(A3–G3)** and 1.00 μm (magnification 50,000×) in **(A4–G4)**. The white arrow denotes the most prominent wax crack.

To elucidate the crystal morphology of the 35 pear cultivars, wax structures were detected by FESEM. Interestingly, the wax morphology showed various amorphous structures in different cultivars, including crystalline plates, irregular ovate crystals, platelets and rodlets with wax crystals (**Figure 3** and Supplementary Figures S4, S5). For example, the mature fruits of ‘Qiubai’, ‘Kuerle’, ‘Clapp Favorite’ and ‘Jinfeng’ were covered by wax with irregular ovate crystals (**Figures 3D1–D4** and Supplementary Figures S4C1–C4,I1–I4, S5I1–I4), whereas the fruit wax of ‘Chili’, ‘Eli’, ‘Hongnanguo’, ‘Balixiang’, ‘Lüamute’, ‘Abbe Fetel’, ‘Xueqing’ and ‘Yuluxiang’ were composed of numerous transversely polygonal rodlets and platelet crystals (**Figures 3A1–A4** and Supplementary Figures S4A1–A4,G1–G4,H1–H4,K1–K4, S5C1–C4,L1–L4,O1–O4), and glossy transversely polygonal rodlet wax covered the fruit surfaces of the cultivars including ‘Zhongli No. 2’ and ‘Pingguoli’ (**Figures 3G1–G4** and Supplementary Figures S5D1–D4). The mature fruits of ‘Kucheamute’ and ‘Hongxiangsu’ were covered by wax crystals with vertically polygonal rodlets with numerous platelets (**Figures 3C1–C4,G1–G4**), and ‘Nanguoli,’ ‘Huagai’ and ‘Xizilü’ were covered by wax crystals with glossy vertically polygonal rodlets (**Figures 3B1–B4** and Supplementary Figures S4E1–E4,S5K1–K4). Remarkably, on the fruit surfaces of ‘Zhongli No. 2’, ‘Eli’, ‘Abbe Fetel’, ‘Bartlett Max Red’ and ‘Yuluxiang’, these structures separated from the wax layer, producing cracks and discontinuity in the outer layer of wax (**Figures 3G1–G4** and Supplementary Figures S4A1–A4, S5C1–C4,D1–D4,O1–O4).

It is generally believed that the various epicuticular wax crystal types, such as rodlets, tubules and platelets, deriving from self-assembly processes, were mainly based on their different chemical compositions (Chu et al., 2017). It has been reported that the tubule-shaped wax crystal is mainly determined by higher proportions of secondary alcohol tubes and β-diketone wax components in blueberry fruits (Chu et al., 2017), and the platelet wax crystals are caused by a higher proportion of aldehydes and alkanes in the epicuticular waxes of ‘Newhall’ navel orange fruits (Wang et al., 2014). However, it seems that similar wax structures can be formed by different chemical compositions in different species. For example, platelets are the most common wax structures, and wax platelets in *Sedum rupestre* are characterized by a high amount of triterpenoids, whereas platelets found in the Poaceae (e.g., Triticum, Zea) are generally dominated by primary alcohols (Stevens et al., 1995; Koch et al., 2006). However, many diverse structural and intermediate forms, environmental effects, and erosion of the waxes lead to undetermined shapes (Koch and Ensikat, 2008). Alkanes and primary alcohols were the dominant compounds of the epicuticular wax of pear fruits. The epicuticular wax had an amorphous structure in 35 pear cultivars, which makes classification of the pear fruits unclear. This denotes that wax structures in pear species are not only based on their dominant chemical composition but also influenced by other factors. It has been reported that wax structures on plant surfaces could be influenced by the molecular order (crystallinity) and orientation of the polar groups (polarity) of cutin, and the resulting template effects of the substrates could be used to influence the orientation and preferred direction of crystal growth (Koch et al., 2006). Given the present lack of knowledge about the molecular order and polarity of the cutin network, the factors contributing to the growth of wax structures on the cuticles of pear fruits still need further study.

### The Cluster Analysis of the 35 Pear Cultivars

**FIGURE 4:**
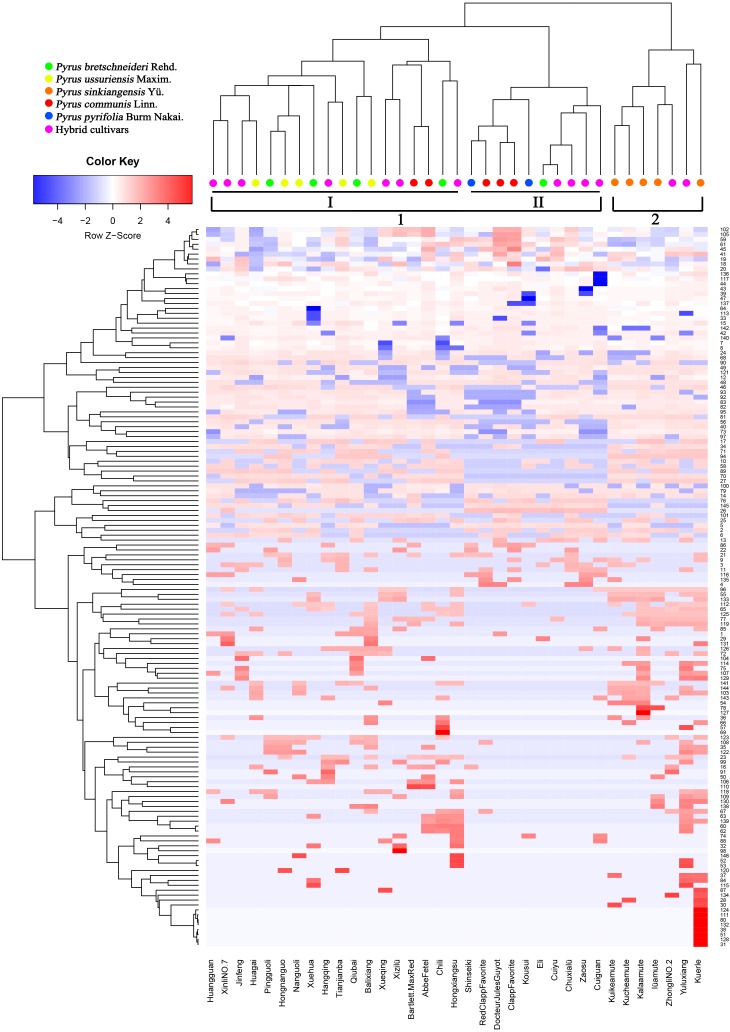
Heatmap and clustering of 146 wax components in 35 pear cultivars. Colors indicate chemical composition levels. The numbers of each row represent the wax component numbers corresponding to the accession numbers in Supplementary Table S1. Each column represents a pear cultivar.

Prior studies have noted that various chemical compositions of epicuticular wax, such as alkanes (Li et al., 2013), terpenes (Medina et al., 2006), flavonoids (Essokne et al., 2012), and fatty acids (Velasco and Goffman, 1999), could be a useful taxonomic marker for classifying the family, genus or species in higher plants and can reflect both ecological and genetic relationships. To elucidate epicuticular wax relationships among 35 pear cultivars, the concentrations of 146 epicuticular wax compounds found in the mature fruits of 35 pear cultivars were clustered through heatmap analysis (**Figure 4**). The dendrogram generated from the heatmap of wax composition could be divided into two major groups (**Figure 4**). Group one, including *P. bretschneideri*, *P. ussuriensis*, *P. communis*, *P. pyrifolia* and the hybrid cultivars, could be further divided into two subgroups (I–II). Subgroup (I) contains all five *P. ussuriensis*, four *P. bretschneideri*, seven hybrid cultivars and two *P. communis* cultivars, whereas the remaining three *P. communis*, one *P. bretschneideri*, two *P. pyrifolia* and four hybrid cultivars were clustered into Subgroup (II). The close genetic relationships between *P. bretschneideri*, *P. ussuriensis*, and *P. pyrifolia* based on our data are consistent with previous results using isozymes, SSR markers and RAPD markers (Bao et al., 2007). Group two was composed of *P. sinkiangensis* and two hybrid cultivars. All five cultivars of *P. sinkiangensis* were grouped into the same clade (**Figure 4**), indicating that the components and contents of their epicuticular wax are similar, and their relationships are phylogenetically close. In addition, ‘Yuluxiang’ and ‘Kuerle’ were clustered into group two (**Figure 4**). Though ‘Yuluxiang’ is a hybrid cultivar of ‘Kuerle’ (*P. ussuriensis*) and ‘Xuehua’ (*P. bretschneideri*), the results of this study indicate that the ‘Yuluxiang’ pear is much more phylogenetically close to the *P. sinkiangensis* from the hereditary perspective of epicuticular wax. The chemical composition of two major groups (group one and two) and two subgroups (I–II) were compared with t-test. We found that the statistical significant number have 60 and 62 according to two major groups and two subgroups, respectively (Supplementary Table S3).

### Principal Component Analysis of Wax Compounds

**FIGURE 5:**
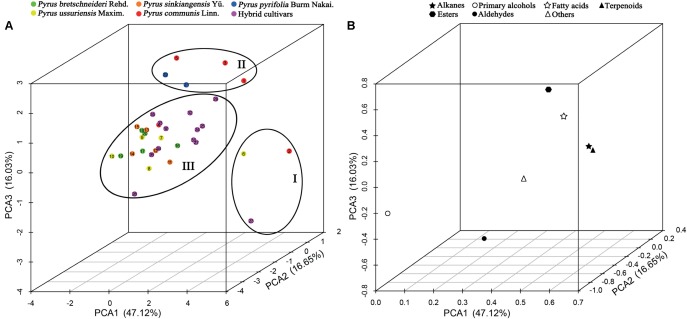
PCA of seven main classes of wax compounds in 35 pear cultivars. **(A)** Scatter plot of PCA scores and **(B)** loadings plot of the PCA. The numbers in **(A)** correspond to the sample numbers given in **Table 1**. Percentages in parentheses are the variance of each component.

The seven classes of epicuticular wax compounds (alkanes, primary alcohols, fatty acids, aldehydes, esters, terpenoids, and others) found in 35 pear cultivars were analyzed through principal component analysis (PCA). The scatter plot of the scores of the PCs of 35 pear cultivars and the corresponding loadings plot of epicuticular wax compounds are shown in **Figure 5**. PCA1 (47.12%), PCA2 (16.65%) and PCA3 (16.03%) accounted for 79.80% of the total variance, which was high enough to represent all the variables (**Figure 5**). The 35 pear cultivars could be divided into three groups on the basis of the relationships between cultivars (scores) and their seven classes of wax compounds (loadings). Group one was on the positive side of PCA1 with the longest distance and on the negative side of PCA3 and included cultivars ‘Jinfeng’, ‘Tianjianba’ and ‘Clapp Favorite’, which were characterized by high concentrations of alkanes (**Figure 5AI**). Cultivars on the positive side of PCA2 with the longest distance formed the second group and contained five pear cultivars (‘Kousui’, ‘Docteur Jules Guyot’, ‘Shinseiki’, ‘Bartlett MaxRed’ and ‘Abbe Fetel’); these cultivars were characterized as cultivars with lower concentrations of terpenoids (**Figure 5AII**). The third group contained all the other cultivars, and there was no consistency with respect to the composition of wax compounds (**Figure 5AIII**).

To further understand how pear cultivars were affected by cuticular wax compositions, we performed the PCA analysis using 146 wax compounds. Compared with the former PCA analysis using seven classes of epicuticular wax compounds, different similar result showed that the PCA1 (64.45%), PCA2 (8.12%) and PCA3 (6.40%), explained 78.97% of the variation (Supplementary Figure S6). However, the 35 pear cultivars could be divided into five groups based on the relationships between cultivars (scores) and their 146 wax compounds (loadings). ‘Clapp Favorite’ formed the first group, which were characterized by high concentrations of octacosane. Group two contained six pear cultivars (‘Bartlett Max Red’, ‘Hongnanguo’, ‘Nanguoli’, ‘Huagai’, ‘Xuehuali’ and ‘Zhongli No. 2’), which were characterized with high concentrations of triacontane-1,30-diol. Group three including two cultivars (‘Eli’ and ‘Huangguan’), which were mainly characterized by high concentrations of triacontane. ‘Abbe Fetel’, ‘Hongxiangsu’ and ‘Xueqing’ characterized with lack of triacontane and hentriacontane were clustered into group four. The fifth group contained all the other cultivars, and these cultivars were characterized with high concentrations of hentriacontane (Supplementary Figure S6A).

## Conclusion

The total wax content of mature fruits was highly variable among 35 pear cultivars, ranging from a high of 2.44 ± 0.41 mg/cm^2^ (‘Docteur Jules Guyot’, *P. communis*) to a low of 0.46 ± 0.03 mg/cm^2^ (‘Huagai’, *P. ussuriensis*). A total of 146 epicuticular wax compounds were detected through GC-MS analysis. Although the composition and concentration of wax compounds varied greatly, alkanes (422.73 ± 205.79 μg/cm^2^, 40.72 ± 12.03%), primary alcohols (271.06 ± 210.96 μg/cm^2^, 24.48 ± 10.57%) and terpenoids (116.39 ± 98.72 μg/cm^2^, 11.80 ± 8.76%) were the predominant wax compounds in all 35 pear cultivars. FESEM images showed that epicuticular wax crystals of the 35 pear cultivars were mostly amorphous structures, including crystalline plates, irregular ovate crystals, platelets and rodlets. The cluster analysis of wax composition and concentration revealed that the *Pyrus bretschneideri* cultivars were grouped much closer to *Pyrus pyrifolia* and *Pyrus ussuriensis*, whereas *Pyrus sinkiangensis* were clustered into a distant separate group. The 35 pear cultivars could be divided into three groups and five groups based on the principal component analysis (PCA) using seven main classes of epicuticular wax compounds and all of the 146 wax compounds, respectively. Taken together, ‘Docteur Jules Guyot’ (*P. communis*), ‘Tianjianba’ (*P. ussuriensis*), ‘Kuerle’ (*Pyrus sinkiangensis* Yü.), ‘Qiubai’ (*P. bretschneideri*) and ‘Shinseiki’ (*P. pyrifolia*) should be the most representative parent cultivars for the future pear breeding with higher wax concentrations.

## Author Contributions

HY and SZ: conceived and designed the experiments. XW, YC, GW, and PC: performed the experiments. XW and YC: analyzed the data. ZS and KQ: contributed reagents, materials, analysis tools or data. XW, XQ, HY, and SZ: wrote the paper.

## Conflict of Interest Statement

The authors declare that the research was conducted in the absence of any commercial or financial relationships that could be construed as a potential conflict of interest.
