# Supplementary material for: Chemical Composition and Crystal Morphology of Epicuticular Wax in Mature Fruits of 35 Pear (Pyrus spp.) Cultivars
Source: Front Plant Sci. 2018 May 23;9:679. doi: 10.3389/fpls.2018.00679 (PMC5974152; doi:10.3389/fpls.2018.00679)
Supplement: Supplementary file 1 [file Presentation_1.pdf]

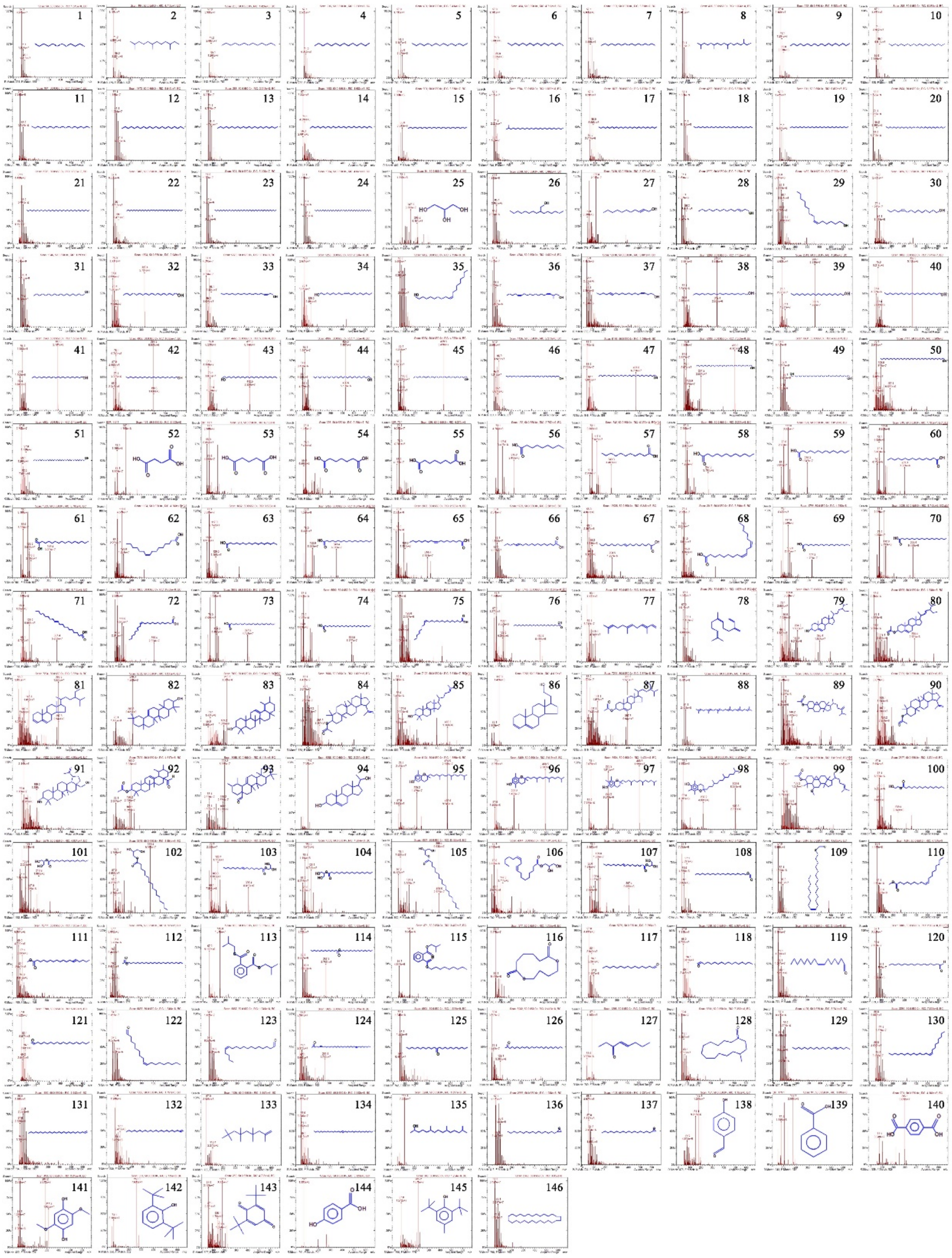

Figure S1. A total of 146 chemical structures were detected and mass spectra of the wax compositions were obtained from the epicuticular wax of 35 pear cultivars.

The numbers correspond to the wax composition numbers given in Table S1.

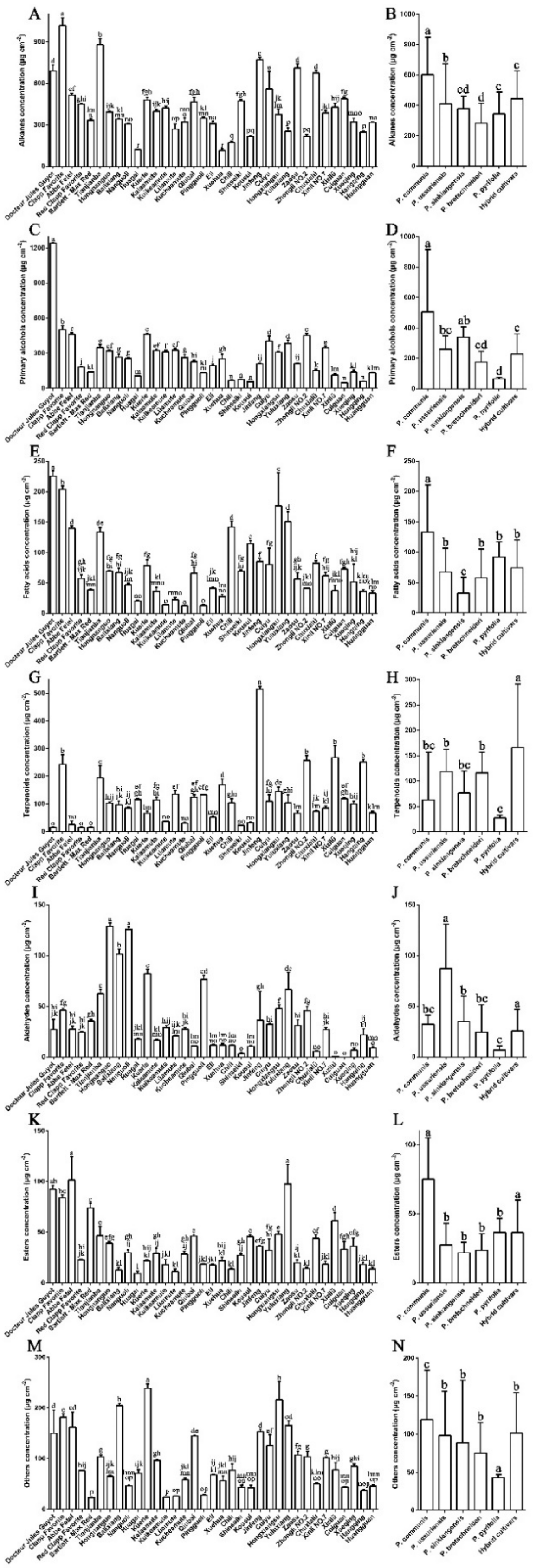

Figure S2. Amounts of seven main classes of wax compounds in mature fruits of 35 pear cultivars and among six cultivated species. A) and B) Alkanes; C) and D) primary alcohols; E) and F) fatty acids; G) and H) terpenoids; I) and J) aldehydes; K) and L) esters; M) and N) others.

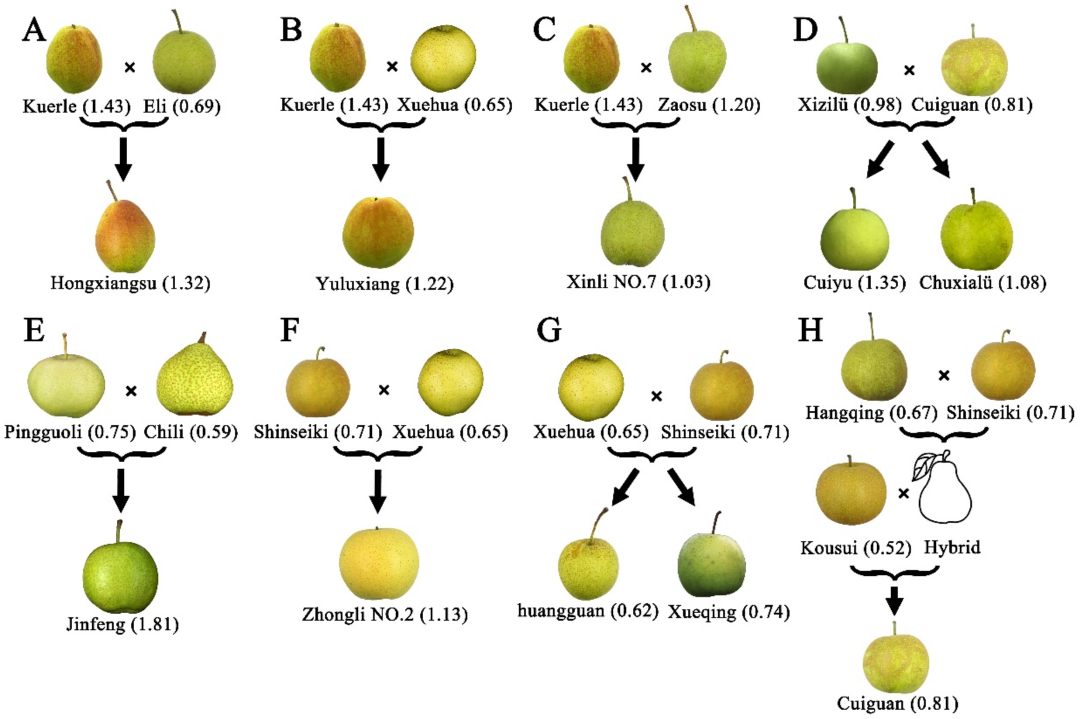

Figure S3. The epicuticular wax concentrations of 10 hybrid combinations of 20 pear cultivars. A) ‘Hongxiangsu’; B) ‘Yuluxiang’; C) ‘Xinli NO.7’; D) ‘Cuiyu’ and ‘Chuxialü’; E) ‘Jinfeng’; F) ‘Zhongli No. 2’; G) ‘Huangguan’ and ‘Xueqing’ and H) ‘Cuiguan’. The number in the bracket represents wax concentration, and the unit is mg/cm^2.

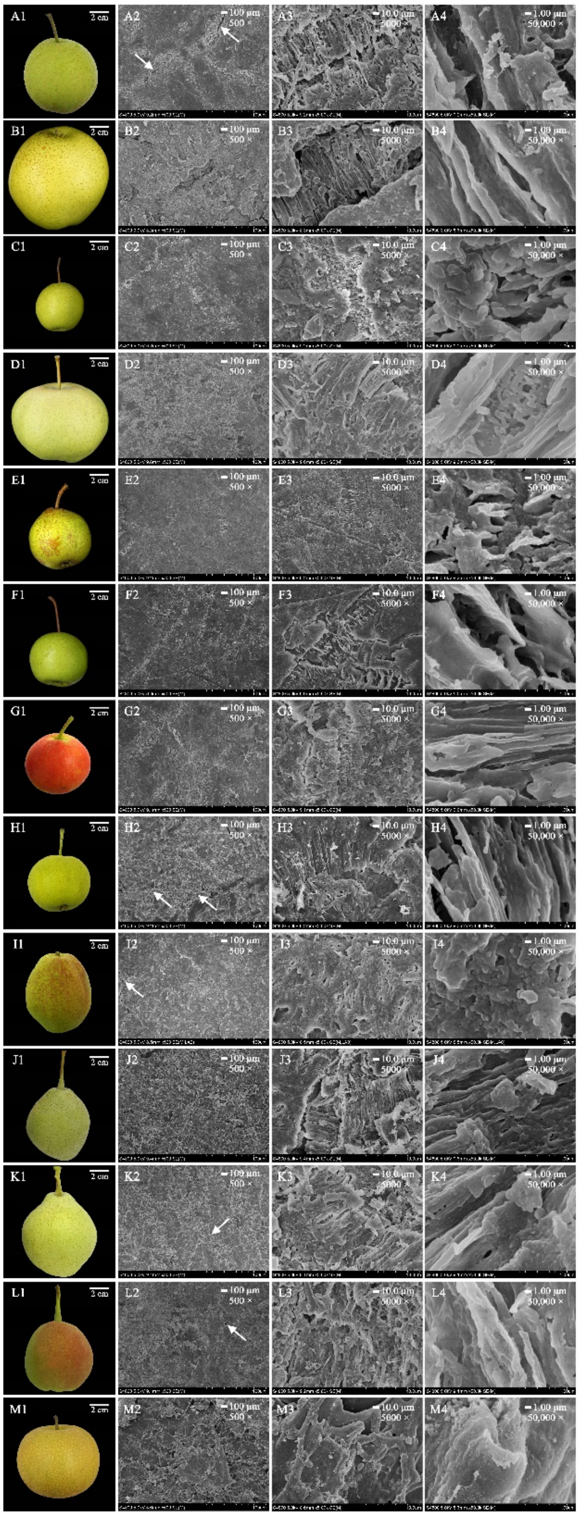

Figure S4. The morphology of mature fruits and the magnification series of the FESEM images of epicuticular wax in 13 pear cultivars.

A1-M1 are mature fruits of ‘Eli’, ‘Xuehua’, ‘Qiubai’, ‘Pingguoli’, ‘Tianjianba’, ‘Huagai’, ‘Hongnanguo’, ‘Balixiang’, ‘Kuerle’, ‘Kalaamute’, ‘Lüamute’, ‘Kuikeamute’ and ‘Kousui’. Scale bars represent 100 μm (magnification 500×) in A2- M2, 10.0 μm (magnification 5000×) in A3-M3 and 1.00 μm (magnification 50,000×) in A4-M4. The white arrow denotes the most prominent wax crack.

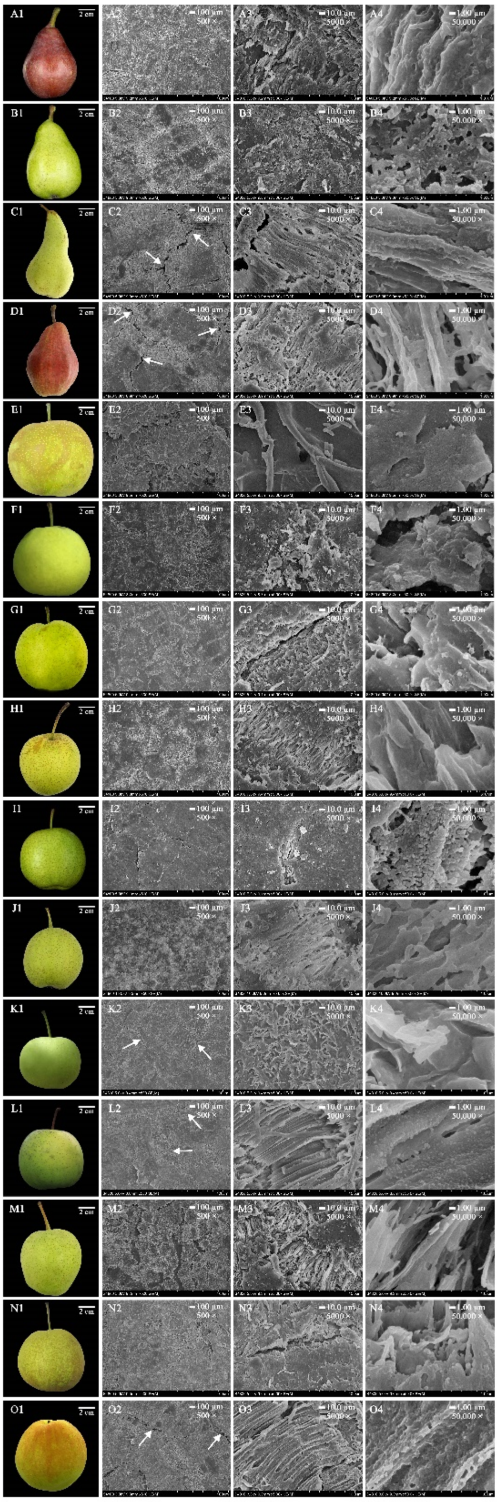

Figure S5. The morphology of mature fruits and the magnification series of the FESEM images of epicuticular wax in 15 pear cultivars.

A1-O1 are mature fruit of ‘Red Clapp Favorite’, ‘Docteur Jules Guyot’, ‘Abbe Fetel’, ‘Bartlett Max Red’, ‘Cuiguan’, ‘Cuiyu’, ‘Chuxialü’, ‘Huangguan’, ‘Jinfeng’, ‘Xinli NO.7’, ‘Xizilü’, ‘Xueqing’, ‘Zaosu’, ‘Hangqing’ and ‘Yuluxiang’. Scale bars represent 100 μm (magnification 500×) in A2- O2, 10.0 μm (magnification 5000×) in A3-O3 and 1.00 μm (magnification 50,000×) in A4-O4. The white arrow denotes the most prominent wax crack.

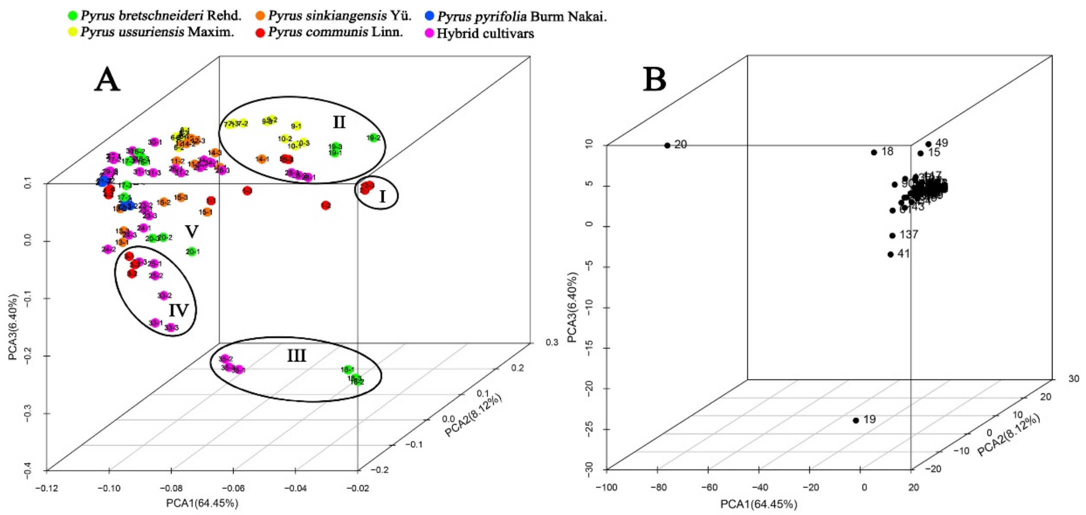

Figure S6. PCA of 146 wax compounds in 35 pear cultivars. (A) Scatter plot of PCA scores and (B) loadings plot of the PCA. The numbers in (A) correspond to the sample numbers given in Table 1. Percentages in parentheses are the variance of each component.
